# Supplementary material for: Predicting Distribution of the Asian Longhorned Beetle, Anoplophora glabripennis (Coleoptera: Cerambycidae) and Its Natural Enemies in China
Source: Insects. 2022 Jul 29;13(8):687. doi: 10.3390/insects13080687 (PMC9409243; doi:10.3390/insects13080687)
Supplement: Supplementary file 1 [file insects-13-00687-s001.zip › insects-1809327-supplementary.pdf]

Table S1. Environment variables for *Anoplophora glabripennis* distribution model

Environment variable	Variable type
Bio_1	Annual mean temperature/℃
Bio_9	Annual precipitation/mm
Bio_14	Precipitation of the driest period/mm
Bio_15	Precipitation seasonality (CV)
Bio_20	Aspect
Bio_21	Elevation
Bio_23	gm_lc
Bio_25	veg

Table S2. Environment variables for *Dastarcus helophoroides* distribution model

Environment variable	Variable type
Bio_1	Annual mean temperature/℃
Bio_6	Minimum temperature of the coldest month/℃
Bio_8	Mean temperature of the wettest quarter/℃
Bio_11	Mean temperature of the coldest quarter/℃
Bio_12	Annual precipitation/mm
Bio_21	Elevation
Bio_23	gm_lc
Bio_25	veg

Table S3. Environment variables for *Dendrocopos major* distribution model

Environment variable	Variable type
Bio_6	Minimum temperature of the coldest month/℃
Bio_9	Annual precipitation/mm
Bio_11	Mean temperature of the coldest quarter/℃
Bio_12	Annual precipitation/mm
Bio_19	Precipitation of the coldest quarter/mm
Bio_21	Elevation
Bio_23	gm_lc
Bio_25	veg

Table S4. Proportion of suitable area of *Anoplophora glabripennis* in China

	Suitable area		Unsuitable area	
	Area (×10^4 km^2)	Proportion (%)	Area (×10^4 km^2)	Proportion (%)
Current	55.85	5.82	904.15	94.18
2050	60.50	6.29	899.50	93.70
2090	57.29	5.95	902.71	94.03

Table S5. Proportion of suitable area of *Dastarcus helophoroides* in China

	Suitable area		Unsuitable area	
	Area (×10^4 km^2)	Proportion (%)	Area (×10^4 km^2)	Proportion (%)
Current	321.14	33.45	638.86	66.55
2050	340.82	35.50	619.18	64.50
2090	341.24	35.55	618.76	64.45

Table S6. Proportion of suitable area of *Dendrocopos major* in China

	Suitable area		Unsuitable area	
	Area (×10^4 km^2)	Proportion (%)	Area (×10^4 km^2)	Proportion (%)
Current	340.59	35.48	619.41	64.52
2050	369.67	38.51	590.33	61.49
2090	372.23	38.77	587.77	61.23

Table S7. In current–2050 and 2050–2090 stages, suitable area changes of *Anoplophora glabripennis* in China (×10^4 km^2)

	**Expansion**	**Stability**	**Contraction**	**Unsuitable**
Current-2050	9.8658	50.6519	5.1637	894.3186
2050-2090	1.5829	55.7266	4.7975	897.8931

Table S8. In current–2050 and 2050–2090 stages, suitable area changes of *Dastarcus helophoroides* in China (×10^4 km^2)

	**Expansion**	**Stability**	**Contraction**	**Unsuitable**
Current-2050	30.2153	310.6031	9.9967	609.1849
2050-2090	9.3844	331.9155	8.9187	609.7813

Table S9. In current–2050 and 2050–2090 stages, suitable area changes of *Dendrocopos major* in China (×10^4 km^2)

	**Expansion**	**Stability**	**Contraction**	**Unsuitable**
Current-2050	57.8557	311.8097	28.4732	561.8614
2050-2090	21.1631	351.0842	18.5938	569.1589
